# Syphilis as a Miasm

**Published:** 1888-05

**Authors:** J. T. Kent


HOMŒOPATHIC PHYSICIAN,

A MONTHLY JOURNAL OF

HOMŒOPATHIC MATERIA MEDICA AND CLINICAL MEDICINE.

"If our school ever gives up the strict inductive method of Hahnemann, we are lost, and deserve only to be mentioned as a caricature in the history of medicine."—CONSTANTINE HERING.

Vol. VIII. MAY, 1888. No. 5.

### SYPHILIS AS A MIASM.

(Notes from an extemporaneous lecture by Professor J. T. Kent.)

It is difficult to know where to begin and where to end the discussion of syphilis—how much to say, or what to leave unsaid. Volumes have been written upon syphilis. Some are worth reading, others are not.

It doesn't help you to cure your patient or to understand the nature of the disease to go back and try to discover the first case of syphilis. You will gain nothing by supposing that it originated among the North American Indians, that it was a product of the French Revolution, or that it has been transmitted through many generations to ours from the remotest ages. It is sufficient to know that the disease exists. It is not in my department to give you its history or its diagnostic relations, but only to consider it as a miasm.

One important drawback to the study of syphilis is the fact that the disease as it comes to us through the books is always under allopathic treatment. Fox has given us some good points. Bumstead was no doubt a great syphilographer.

These writers have described the beginning, the course, and what they consider as the end of syphilis; but a large number of their cases were under allopathic treatment, and the result is that they have reported what they call relapses which are studied as relapses, *i. e.*, returns of the old disease.

Studied under homœopathic treatment, the disease presents an entirely different aspect, as different as day is from night.

The study of cases *without treatment* is what we need. But of such we have very few. Allopathy modifies the disease in that it suppresses its manifestations. Study Bumstead, and you will find pictured there its worst forms and complications, but you will not find the correct form. The view is biased. There is no work in allopathy or Homœopathy to-day that gives a correct view!

In allopathy, as soon as the chancre appears, it is cauterized; then the glands are affected, and buboes follow; maculæ appear; ulcers appear in the throat, and these are immediately cauterized, after which the hair falls out.

What would be the result if the chancre were let alone? Then we could see the true nature of the disease. We could see whether it tends to run a certain course and recover, and to what extent this lifelong miasm is due to suppression; whether it is altogether the result of suppression. Syphilis must run a certain course.

It often begins with chills and bone pains. About the fifteenth day the chancre appears. This is the first effort of nature to cure. This eruption is suppressed by allopathic treatment just as the psoric eruption is suppressed, and miasm is the result. Thus syphilis, being a constitutional disease, is made, I may say, ten times more constitutional by suppression. The disease is thrown back on the nervous system, the nerve force is perverted, and a vicarious expenditure takes place. Often with the chancre come the buboes. These are treated with Iodine and various ointments. Does this tend to throw out the disease? No! It aggravates it tenfold.

Next come eruptions on the skin. Local applications are immediately resorted to to suppress them.

Are they treated scientifically? I say No! *Hahnemann* says No!

Then come ulcers in the throat. Are they treated with an idea of their cause? Are they allowed to evolve themselves? No! They are immediately driven back! At every place the disease is refused its own expressions!

When the hair falls out, lotions are applied to the head to stimulate the hair follicles to hold their sprouts.

This is the course of treatment we find laid out in allopathic literature.

Under homœopathic treatment the course is very different.

A patient comes to you with a chancre. Instead of cauterizing it let it alone. But the patient says, "It must be cauterized—it is the old way." Do not do it.

What if it increases and remains for two or three months? All right, that is what you expect. You are afraid the young man will go off and leave you? That has not been my experience.

The sore is painless. Tell him that if you cauterize it you will only make his condition worse. If you do not cauterize it, in the course of a few months you can restore him to health; he will be cured and will transmit no taint to his children. If some leave you others will not, and you will have enough remain to be cured.

Study the totality of symptoms and select your remedy.

Under the action of this proper remedy the sore becomes soft and commences to discharge enormously, instead of getting harder.

The standard authorities state that the bubo has little tendency to suppurate; but under homœopathic treatment it often suppurates, as this is the easiest way for nature to rid the system of the disease.

Thus Homœopathy changes the entire aspect of the disease from its beginning. Now the eruptions come to the skin, and you will probably have to change your remedy. Then, by the right prescription, you cure the disease from within. The eruption is less though the stage goes on. You overcome the cause. You use no local applications, no washes or unguents. The sore which first appears is the last to heal. By this (which is in accordance with the law that, "diseases get well in the reverse order of their coming") you know that the patient is getting well.

Now watch; the sore throat will appear. If you don't see the remedy the first day, have him call again in a few days. Watch his condition, the course the ulcer is taking, its color and direction. For a remedy to conform to all these things you must go to the *Materia Medica.* Again in the throat, the first patch that comes is the last to heal. The day you give the right remedy that day the ulcer ceases to enlarge, molecular death stops. When the last ulcer is gone it will return no more as long as he lives.

The next stage is the falling of the hair. Notice where it commences, and get the entire list of remedies that have falling of the hair, also a list of syphilitic remedies, and compare; but most of all the entire range of proved remedies. Use no Fowler's solution, no cantharis, no colognes.

Now, what do we see? It is a strange fact that the sooner one stage is cured, the sooner the next one comes on. In six months you may carry the patient through all the stages and cure him. The shadow of all the stages will present the guiding images for you to select your remedies from. Destroy no symptoms.

Hahnemann made the mistake, and many homœopaths have done likewise, of not distinguishing between chancroid and chancre, which fact accounts for some of his reports of cases cured very speedily with Merc.$^{30}$. The distinction between chancroid and chancre had not been made in Hahnemann's day. The cures with a dose of Mercury are not cures of the syphilitic miasm.

If you take syphilis, as abused by allopathic treatment, and attempt to adjust homœopathic therapeutics to it, you will fail. Hence the bugbear that syphilis has been to homœopaths. This should not be so.

There is a peculiar fact about the contagion of syphilis (it is true of sycosis also) that you will not find in accepted literature. 'Tis that the person infected takes the disease in the stage which it is in at the time of infection. He does not go back and have the disease from the beginning, but finishes it from the stage which was present in the person who spread the infection. This fact I have learned from observation in my own practice. Many wives suffer from the third stage who never have had the first, and but a shadow of the second.

A young girl was betrothed to a young man who was suffering from a relapse. He had ulcers in his mouth, secondary form of syphilis. One day she happened to eat out of the same spoon which this young man had been using. The result was she took the disease, and came out with ulcers in mouth and throat. Her lover, very much alarmed, came and told me the circumstances. I watched that case, and saw her pass through the symptoms of secondary syphilis with ulcers in the throat, and finally lose her hair. But she never had a chancre or a bubo or eruption on the skin. I have seen several similar cases which proved to me this principle of contagion.

The general course of syphilis is:

I. Primary Stage.

- 1st. Chancre.

- 2d. Buboes.

II. Secondary Stage.

- 3d. Skin affections, maculæ, and other eruptions.

- 4th. Ulcers in the throat.

- 5th. Loss of hair.

III. Tertiary.—Nerve and bone affections.

The disease may be suppressed in the first stage and remain latent for some time.

A young man came to me who had been sickly for eighteen months. His symptoms called for Kali-iod. I put him on Kali-iod., and in a short time he broke out with a syphilitic eruption.

I knew there was nothing in *Kali-iod.* which would produce such an eruption as that, and on closer investigation of the case, I found that he had had a chancre which had been suppressed by large doses of Merc., and the resulting syphilitic miasm was what had been making him sick. The Iodide of Potassium had antidoted the Mercury and the miasm had come out.

Any miasm may be suppressed and held latent and not show itself as such; but *when the miasm does not appear in symptoms* the person is sickly. In such cases it is often difficult to get symptoms to prescribe on. The lazy doctor will not be able to find any; but by *close investigation* you can generally find some symptoms. If he really has no symptoms his case is generally incurable, since curable diseases express themselves by signs and symptoms.

If he is really incurable, the symptoms of the disease have been driven back so deep that he has only an undefined sense of feeling badly. In the last stages, when the patient has "been the rounds" of allopathic suppression, he returns from the Hot Springs and comes to you, perhaps too late. He suffers from the category of nerve syphilis. He has bi-parietal pains, exostosis, thickening of the periosteum. We do not know that we would have such forms if it were not for suppression.

Under homœopathic treatment, although the patient taken in the primary stage does not get well without a shadowing at least of the secondary stage, yet of the tertiary forms the shadow is so slight that we cannot really say that they exist at all.

When syphilis attacks the nerve centres, we have softening of the brain, brain tumors, and death. How much of the nerve disease is due to suppression we cannot now determine. These tertiary forms never get well unless you can bring them back into the secondary stages.

A patient who had been under allopathic care for many years, who had taken Iodine, Bromide, Corrosive sublimate, Iodide of Potassium, etc., in large quantities, came to me in the last stages of syphilis with agonizing head pains. I prescribed for him, he became weak minded, but the pains all left him. In the further treatment of the case what condition did I find next? What could we expect to find according to the law of direction? Loss of hair and then ulcers in the throat—sure enough, such was the case. The loss of hair and the ulcers worried him so much, that he went away and left me.

The case is none the less valuable, however, since it serves to illustrate the manner in which the disease may get well even when in the last stages.

You can see now the nature of the disease.

Under homœopathic treatment, though I have had many cases, I have *never seen a relapse;* while allopaths report relapses in a large percent. of their cases.

You ask me to outline the treatment, which would necessitate my going into the numerous forms and groups of skin symptoms and nerve manifestations of this miasm. Volumes might be written on this subject and in the end you could only be directed to study well the *Materia Medica,* and treasure up no names to arrange medicines for. Take the case as though you had never heard of such a set of symptoms in a sick man, but were perfectly acquainted with such symptoms in provings, remembering that the pathognomonic symptoms are not the ones you shall need the likeness of, but the uncommon ones. Destroy no symptoms that nature has sent out to guide you to your remedies. Some patients will leave you, but if you are acquainted with the art of healing, you will have all you can attend to among the faithful and intelligent members of your cities and villages.

